# Heart Failure Among Asian American Subpopulations

**DOI:** 10.1001/jamanetworkopen.2024.35672

**Published:** 2024-09-26

**Authors:** Yan Cheng, Adrienne N. Poon, Youxuan Ling, Wen-Chih Wu, Ali Ahmed, Tadas S. Vasaitis, Gurusher Panjrath, Mark Edberg, Mardi Gomberg-Maitland, Ying Yin, Stuart J. Nelson, Qing Zeng-Treitler

**Affiliations:** 1 Department of Clinical Research and Leadership, George Washington University, Washington, DC; 2 Veterans Affairs (VA) Medical Center, Washington, DC; 3 Department of Medicine, George Washington University, Washington, DC; 4 Department of Medicine and Department of Epidemiology, Brown University, Providence, Rhode Island; 5 Long Term Care Service and Support Center of Innovation, VA Medical Center, Providence, Rhode Island; 6 School of Pharmacy and Health Professions, University of Maryland Eastern Shore, Princess Anne, Maryland; 7 Department of Prevention and Community Health, George Washington University, Washington, DC

## Abstract

**Importance:**

Heart failure (HF) is a leading cause of death in the US. The current evidence on the burdens of HF in Asian American populations, especially Asian American subgroups, is limited and inconsistent.

**Objective:**

To assess and compare the incidence and prevalence of HF in Asian American subgroups.

**Design, Setting, and Participants:**

This retrospective cohort study used electronic health record data from patients 40 years or older with health care encounters from January 1, 2015, to December 31, 2019, recorded in the Oracle Electronic Health Record Real-World Data database, which has more than 100 health care systems across the US contributing to the database as of February 2024. For prevalence analysis, the study samples were those who had at least 1 encounter in the study calendar year. For incidence analysis, participants were additionally limited to those without HF before the study year who also had encounter(s) the year before the study year. Data analysis was performed from August 1, 2023, to July 31, 2024.

**Exposure:**

Race and ethnicity were determined using patient self-reported data, which were categorized as Black, East Asian, South Asian, Southeast Asian, other Asian (without specified ethnicity), and White.

**Main Outcomes and Measures:**

Outcomes were incidence and prevalence of HF, identified using recorded *International Classification of Diseases, Ninth Revision, Clinical Modification* and *International Statistical Classification of Diseases, Tenth Revision, Clinical Modification* codes. Age- and sex-standardized incidence and prevalence were used to calculate the risk ratio of each racial and ethnic group compared with White patients.

**Results:**

Incidence and prevalence analyses were performed for 6 845 791 patients (mean [SD] age, 62.1 [12.5] years; 59.9% female; 2.8% Asian, 6.7% Black, and 90.5% White) and for 13 440 234 patients (mean [SD] age, 61.7 [12.7] years; 57.0% female; 2.9% Asian, 7.1% Black, and 90.0% White), respectively. Using the 2015 population as the standard, age- and sex-standardized HF incidence was 2.26% (95% CI, 2.07%-2.45%) for Southeast Asian patients, 1.56% (95% CI, 1.31%-1.82%) for South Asian patients, and 1.22% (95% CI, 1.06%-1.38%) for East Asian patients compared with 1.58% (95% CI, 1.57%-1.59%) for White patients and 2.39% (95% CI, 2.36%-2.42%) for Black patients. Similarly, heterogeneous rates in Asian American subgroups were also observed in the prevalence analysis.

**Conclusions and Relevance:**

In this study of HF outcomes, the disparities between Southeast and East Asian patients were larger than those between Black and White patients, with the estimates in Southeast Asian patients being similar to those of Black patients. These findings reinforce that individual Asian ethnicities and cardiovascular risk factors should be considered in the assessment of HF risks.

## Key Points

**Question:**

Given the social, economic, and genetic diversity within the Asian American community, do disparities exist in heart failure rates among different ethnic groups?

**Findings:**

In this cohort study, incidence analysis was performed for 6 845 791 patients and prevalence analysis for 13 440 234 patients, revealing that incidence and prevalence of heart failure varied among Asian American populations. The disparity between Southeast and East Asian patients was more pronounced than the difference observed between Black and White patients, with heart failure rates among Southeast Asian patients comparable to those among Black patients.

**Meaning:**

In the assessment of heart failure risks, Asian American patients should not be regarded as a monolithic group, and individual Asian ethnicities and cardiovascular risk factors should be considered.

## Teaser

This cohort study investigates the incidence and prevalence of heart failure among Asian American populations.

## Introduction

Heart failure (HF) is a leading cause of death in the US, affecting approximately 6.7 million people between 2017 and 2020 and projected to surpass 8 million by 2030.^1^ The lifetime risk is 22.6% in women and 25.3% in men older than 50 years.^2^ Asian American people constitute approximately 7.2% of the US population, totaling 24 million individuals according to 2020 US Census Bureau data.^3^ Given the diverse backgrounds, languages, and cultures of Asian American populations, it is crucial to examine disparities in HF incidence and prevalence among different ethnic groups.

Cardiovascular disease (CVD) is the second leading cause of death among Asian American populations and a major risk factor for HF.^4,5^ Asian American people experience higher premature death rates and slower decreases in heart disease deaths compared with White populations.^6^ Age-adjusted HF rates vary widely across Asia, ranging from 211.86 per 100 000 population in Nepal to 1032.84 per 100 000 population in China.^7^ The prevalence of HF in Asia ranges from 1.3% to 6.7%, with an estimated 1.3% in China, 4.5% in Singapore, and 6.7% in Malaysia.^8,9,10,11,12,13^ The Asian Sudden Cardiac Death in Heart Failure registry reveals regional differences in 1-year mortality, with higher rates in Southeast Asian patients (13%) compared with South Asian (7.5%) and Northeast Asian (7.4%) patients.^14^ These rates suggest underlying distinct genetic and environmental risks for CVD among Asian ethnic groups, influenced by acculturation-related changes in diet and physical activity patterns in the US.^15,16,17^

Data on HF in Asian American populations are limited^18^ and are often presented in aggregated form, overlooking variations in socioeconomic status, immigration history, and environment among different ethnic subgroups. Available data on the burden of HF in Asian American populations are inconsistent. A study by Kaiser Permanente Northern California suggested that “selected outcomes [of HF] for Asian/Pacific Islander and Hispanic patients were more favorable compared with White patients.”^19^ The Office of Minority Health at the US Department of Health and Human Services also reported, “Overall, Asian American adults are less likely than White adults to have heart disease and they are less likely to die from heart disease,”^20^ despite heart disease being the second highest cause of death for Asian American people.^4,5^ Previous work has highlighted a higher CVD burden in the South Asian community^18^ and high HF mortality rates among Indian American populations.^21^ These discrepancies underscore a clear knowledge gap regarding the HF incidence and prevalence in specific Asian American subpopulations.

Large electronic health record (EHR) data offer new avenues for cardiovascular epidemiology research.^22^ To investigate HF among Asian American populations, understanding HF incidence and prevalence using both aggregated and disaggregated data is crucial. We analyzed a comprehensive national EHR dataset that includes composite data from Asian American patients as well as disaggregated data by ethnic subgroups. Our main hypothesis is that disaggregated Asian American subgroups exhibit varying incidence and prevalence of HF compared with the aggregated Asian American group (with unspecified ethnicity). We also included Black and White patients for comparative analysis.

## Methods

### Study Design and Setting

This retrospective cohort study used deidentified data from the Oracle EHR Real-World Data (RWD) database, encompassing records from 108 million patients across more than 100 US health care systems as of February 2024.^23,24^ The study focused on nationwide data from January 1, 2015, to December 31, 2019, to avoid anomalies in data from the COVID-19 pandemic. All data we used are deidentified under the guidance of the data governance council that oversees and monitors Oracle Learning Health Network data use and research, for which patient-level data are not available for public use. Patient consent was waived by the Oracle EHR RWD database because study participants were deidentified. The George Washington University Institutional Review Board reviewed the use of the RWD database in clinical and health services research studies and determined it to be exempt because patient data were extracted from deidentified medical records. The study followed the Strengthening the Reporting of Observational Studies in Epidemiology (STROBE) reporting guideline.

### Study Population

**Figure 1.**
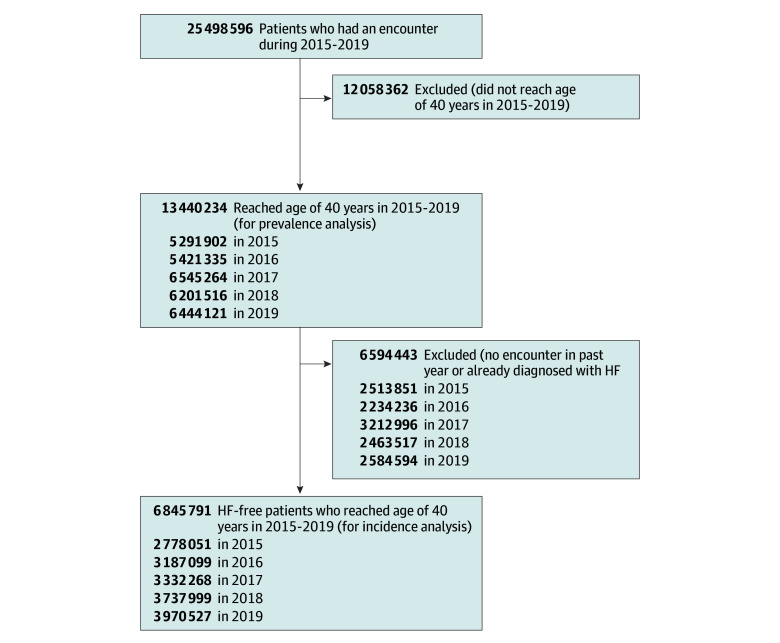
Flowchart of Cohort Selection HF indicates heart failure.

Our study included 25 498 596 active patients having at least 1 encounter from 2015 to 2019 in the Oracle EHR RWD database. In the Chronic Heart Failure Analysis and Registry, the prevalence of HF greatly increased at 40 years and older^25^; therefore, patients 40 years or older (n = 13 440 234) were included to capture the risk of early-onset HF while excluding genetic and congenital causes. Because age and other characteristics of a given patient and the health care systems covered by the data change from year to year, we treated each calendar year as a separate subcohort. For prevalence analysis, patients with at least 1 encounter in a study calendar year were included. For incidence analysis, patients were required to have an encounter preceding the study calendar year and to be free of the study outcome of HF to minimize outcome misclassification. The flowchart for prevalence and incidence analysis is shown in Figure 1.

### Study Outcomes

We defined HF using *International Classification of Diseases, Ninth Revision, Clinical Modification* (*ICD-9-CM*) (428.x, 398.91, 402.01, 402.11, 402.91, 404.01, 404.03, 404.11, 404.13, 404.91, and 404.98) and *International Statistical Classification of Diseases, Tenth Revision, Clinical Modification* (*ICD-10-CM*) (I50.x, I11.0x, I13.0x, and I13.2x) codes, covering the transition of *ICD-9-CM* to *ICD-10-CM* in the US starting October 2015.^26^ Incident HF was diagnosed in patients without prior HF diagnoses, with at least 1 year of history in the database. Prevalent HF included all patients with an HF diagnosis, regardless of history. The incidence and prevalence of HF were calculated by decades of age. Both rates were calculated annually using the following formulas:
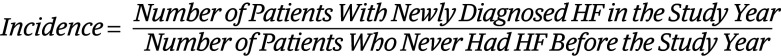
and


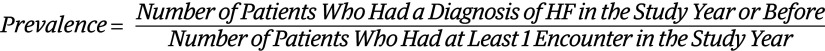
.

The 95% CI for prevalence and incidence was calculated using the formula:
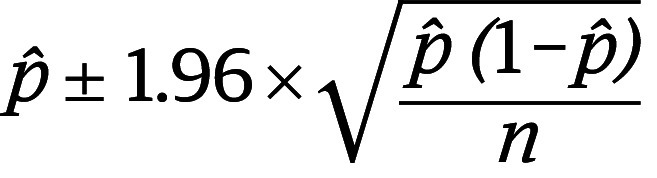
,where *p̂* is the estimate of prevalence or incidence in a study year and *n* is the denominator of prevalence or incidence of the study year. We plotted prevalence and incidence trends by age categories for each racial group.

### Race and Ethnicity Determination

Race was determined using self-reported demographic variables and Centers for Disease Control and Prevention race and ethnicity concept codes.^27^ Race and ethnicity data were self-reported by patients and recorded accordingly. In this study, race included Asian, Black, and White categories. Asian patients were further subgrouped based on regional ethnicity: East Asian (China, Japan, Taiwan, and Korea), South Asian (Afghanistan, Bangladesh, Pakistan, India, Nepal, Bhutan, Maldives, and Sri Lanka), Southeast Asian (Vietnam, Myanmar, Laos, Malaysia, Thailand, Cambodia, Indonesia, Singapore, and Philippines), and other Asian without specified ethnicity. Multiple imputation was not conducted due to missingness of regional ethnicity in approximately 90% of Asian patients. To understand the robustness of estimates, we calculated the incidence and prevalence of HF for the overall population and subgroups of Asian American patients.

### Other Variables

We collected data on self-reported demographics, including age, sex, and marital status. To compare clinical characteristics among race and ethnicity groups, we also identified CVD comorbidities using *ICD-9-CM* and *ICD-10-CM* codes, including coronary heart disease (CHD), acute myocardial infarction (MI), hypertension, diabetes, stroke, and atrial fibrillation.

### Statistical Analysis

We performed descriptive analysis on baseline characteristics, including demographics and medical conditions across racial groups. Continuous variables were described using means (SDs), and categorical variables were presented as numbers (percentages). To ensure comparability of prevalence and incidence estimates across groups, we used 2015 cohorts as a standard population to calculate age- and sex-standardized rates, thereby mitigating bias caused by age and sex differences among groups. Risk ratios (RRs) for each racial group compared with White patients were calculated using the following formula^28^:

*RR* = *p*_1_/*p*_0_,

where *p*_1_ is the age- and sex-standardized rate of a racial group, and *p*_0_ is the age- and sex-standardized rate of White patients. The 95% CI of the RR was calculated as follows: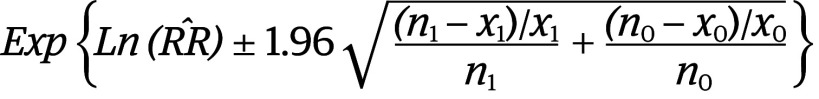,where *n*_1_ is the total sample size of a racial group, *x*_1_ is the number of patients who had the outcome in the group, *n*_0_ is the total sample size of White patients, and *x*_0_ is the number of patients who had the outcome in White patients. We set statistical significance at a 2-tailed *P* < .05. Data analyses were conducted in Python, version 3.7.6 (Python Institute) and R, version 4.0.2 (R Foundation for Statistical Computing). Data analysis was performed from August 1, 2023, to July 31, 2024.

## Results

Incidence analysis was performed for 6 845 791 patients (mean [SD] age, 62.1 [12.5] years; 59.9% female and 40.1% male; 2.8% Asian, 6.7% Black, and 90.5% White) with 17 005 944 episodes of care, with demographic details in Table 1. Ethnicity data revealed high rates of CVD comorbidities in Asian American patients, such as acute MI (8.1% among East Asian patients, 6.1% among South Asian patients, and 12.9% among Southeast Asian patients compared with 3.3% among White patients), diabetes (21.4% among East Asian patients, 33.5% among South Asian patients, and 30.4% among Southeast Asian patients compared with 12.1% among White patients), and hypertension (41.9% among East Asian patients, 45.8% among South Asian patients, and 57.6% in Southeast Asian patients compared with 32.4% among White patients). The prevalence of CHD was 9.3% in South Asian patients vs 6.3% in White patients.

**Table 1.** Baseline Characteristics at Episode Level in the Cohorts of Incidence and Prevalence Analysis, 2015-2019

Characteristic	Patient cohort^a^							
	All	White	Black	All Asian^b^	East Asian	South Asian	Southeast Asian	Other Asian^c^
**Cohort for incidence analysis (n = 6 845 791 unique patients with 17 005 944 episodes)**								
Year								
2015	2 778 051 (16.3)	2 527 371 (16.4)	180 066 (15.9)	70 614 (14.8)	1595 (8.8)	1130 (12.5)	1539 (6.7)	66 349 (15.5)
2016	3 187 099 (18.7)	2 906 306 (18.9)	196 881 (17.4)	83 912 (17.6)	3316 (18.4)	1587 (17.5)	4137 (18.1)	74 870 (17.5)
2017	3 332 268 (19.6)	3 014 633 (19.6)	226 816 (20.0)	90 819 (19.0)	4003 (22.2)	1940 (21.4)	5329 (23.3)	79 541 (18.6)
2018	3 737 999 (22.0)	3 367 908 (21.9)	260 198 (23.0)	109 893 (23.0)	4409 (24.4)	2146 (23.7)	5769 (25.2)	97 561 (22.8)
2019	3 970 527 (23.3)	3 578 311 (23.2)	269 721 (23.8)	122 495 (25.6)	4713 (26.1)	2264 (25.0)	6130 (26.8)	109 368 (25.6)
Age, mean (SD), y	62.1 (12.5)	62.4 (12.5)	58.3 (11.6)	60.2 (12.6)	61.0 (10.0)	57.6 (10.9)	60.0 (9.5)	60.3 (12.9)
Race								
White	15 394 529 (90.5)	NA	NA	NA	NA	NA	NA	NA
Black	1 133 682 (6.7)	NA	NA	NA	NA	NA	NA	NA
Asian	477 733 (2.8)	NA	NA	NA	NA	NA	NA	NA
Sex								
Female	10 190 672 (59.9)	9 163 214 (59.5)	714 860 (63.1)	312 598 (65.4)	11 945 (66.2)	5158 (56.9)	14 600 (63.7)	280 877 (65.7)
Male	6 815 272 (40.1)	6 231 315 (40.5)	418 822 (36.9)	165 135 (34.6)	6091 (33.8)	3909 (43.1)	8304 (36.3)	146 812 (34.3)
Married	10 011 428 (58.9)	9 282 691 (60.3)	403 471 (35.6)	325 266 (68.1)	11 151 (61.8)	7104 (78.4)	11 411 (49.8)	295 597 (69.1)
CHD	1 050 362 (6.2)	973 233 (6.3)	55 418 (4.9)	21 711 (4.5)	915 (5.1)	844 (9.3)	1087 (4.7)	18 865 (4.4)
Acute MI	567 490 (3.3)	508 030 (3.3)	44 226 (3.9)	15 234 (3.2)	1452 (8.1)	554 (6.1)	2947 (12.9)	10 281 (2.4)
Hypertension	5 638 621 (33.2)	4 987 903 (32.4)	485 000 (42.8)	165 718 (34.7)	7553 (41.9)	4151 (45.8)	13 203 (57.6)	140 809 (32.9)
Diabetes	2 151 174 (12.6)	1 860 160 (12.1)	208 411 (18.4)	82 603 (17.3)	3864 (21.4)	3033 (33.5)	6974 (30.4)	68 731 (16.1)
Stroke	251 299 (1.5)	218 041 (1.4)	26 694 (2.4)	6564 (1.4)	488 (2.7)	179 (2.0)	611 (2.7)	5285 (1.2)
Atrial fibrillation	631 125 (3.7)	598 971 (3.9)	21 396 (1.9)	10 758 (2.3)	524 (2.9)	150 (1.7)	692 (3.0)	9392 (2.2)
**Cohort for prevalence analysis (n = 13 440 234 unique patients with 30 015 138 episodes)**								
Year								
2015	5 291 902 (17.6)	4 810 936 (17.8)	344 254 (16.2)	136 712 (15.7)	4882 (14.3)	2198 (13.7)	5252 (12.6)	124 374 (16.0)
2016	5 421 335 (18.1)	4 895 346 (18.1)	376 633 (17.7)	149 356 (17.2)	5234 (15.3)	2705 (16.9)	6481 (15.5)	134 926 (17.3)
2017	6 545 264 (21.8)	5 877 559 (21.8)	481 775 (22.6)	185 930 (21.4)	7309 (21.4)	3360 (21.0)	9185 (22.0)	166 058 (21.3)
2018	6 201 516 (20.7)	5 565 042 (20.6)	442 696 (20.8)	193 778 (22.3)	8313 (24.3)	3722 (23.2)	10 313 (24.7)	171 368 (22.0)
2019	6 555 121 (21.8)	5 867 386 (21.7)	483 291 (22.7)	204 444 (23.5)	8436 (24.7)	4027 (25.1)	10 460 (25.1)	181 431 (23.3)
Age, mean (SD), y	61.7 (12.7)	62.1 (12.7)	58.0 (11.7)	60.2 (12.9)	60.7 (10.9)	57.6 (11.3)	59.6 (10.2)	60.2 (13.1)
Race								
White	27 016 269 (90.0)	NA	NA	NA	NA	NA	NA	NA
Black	2 128 649 (7.1)	NA	NA	NA	NA	NA	NA	NA
Asian	870 220 (2.9)	NA	NA	NA	NA	NA	NA	NA
Sex								
Female	17 096 739 (57.0)	15 302 085 (56.6)	1 253 583 (58.9)	541 071 (62.2)	21 531 (63.0)	8704 (54.4)	25 481 (61.1)	485 264 (62.4)
Male	12 918 399 (43.0)	11 714 184 (43.4)	875 066 (41.1)	329 149 (37.8)	12 643 (37.0)	7308 (45.6)	16 210 (38.9)	292 893 (37.6)
Married	17 060 050 (56.8)	15 764 578 (58.4)	724 968 (34.1)	570 504 (65.6)	20 017 (58.6)	11 630 (72.6)	20 182 (48.4)	518 657 (66.7)
CHD	1 608 870 (5.4)	1 475 568 (5.5)	98 147 (4.6)	35 155 (4.0)	1334 (3.9)	1235 (7.7)	1804 (4.3)	30 782 (4.0)
Acute MI	912 713 (3.0)	813 240 (3.0)	75 935 (3.6)	23 538 (2.7)	1781 (5.2)	811 (5.1)	3583 (8.6)	17 363 (2.2)
Hypertension	7 183 183 (23.9)	6 329 081 (23.4)	642 878 (30.2)	211 224 (24.3)	8941 (26.2)	5020 (31.4)	15 247 (36.6)	182 014 (23.4)
Diabetes	2 827 535 (9.4)	2 437 279 (9.0)	283 933 (13.3)	106 323 (12.2)	4566 (13.4)	3674 (22.9)	8146 (19.5)	89 936 (11.6)
Stroke	354 828 (1.2)	305 915 (1.1)	39 622 (1.9)	9291 (1.1)	563 (1.6)	226 (1.4)	783 (1.9)	7718 (1.0)
Atrial fibrillation	1 061 690 (3.5)	997 332 (3.7)	44 438 (2.1)	19 920 (2.3)	782 (2.3)	276 (1.7)	1225 (2.9)	17 637 (2.3)

Abbreviations: CHD, coronary heart disease; MI, myocardial infarction; NA, not applicable.

^a^ Data are presented as number (percentage) of patients unless otherwise indicated.

^b^ All Asian also includes individuals who identified as West Asian, but this was not reported as a disaggregated group per the current US Census definition of Asian.

^c^ Other Asian indicates no other Asian ethnicity was specified.

Prevalence analysis was performed for 13 440 234 patients (mean [SD] age, 61.7 [12.7] years; 57.0% female and 43.0% male; 2.9% Asian, 7.1% Black, and 90.0% White) with 30 015 138 episodes of care (Table 1). Similar trends in CVD comorbidities in Asian American patients were observed, with higher rates of acute MI (5.2% among East Asian patients, 5.1% among South Asian patients, and 8.6% among Southeast Asian patients compared with 3.0% among White patients), diabetes (13.4% among East Asian patients, 22.9% among South Asian patients, and 19.5% among Southeast Asian patients compared with 9.0% among White patients), and hypertension (26.2% among East Asian patients, 31.4% among South Asian patients, and 36.6% among Southeast Asian patients compared with 23.4% among White patients).

**Figure 2.**
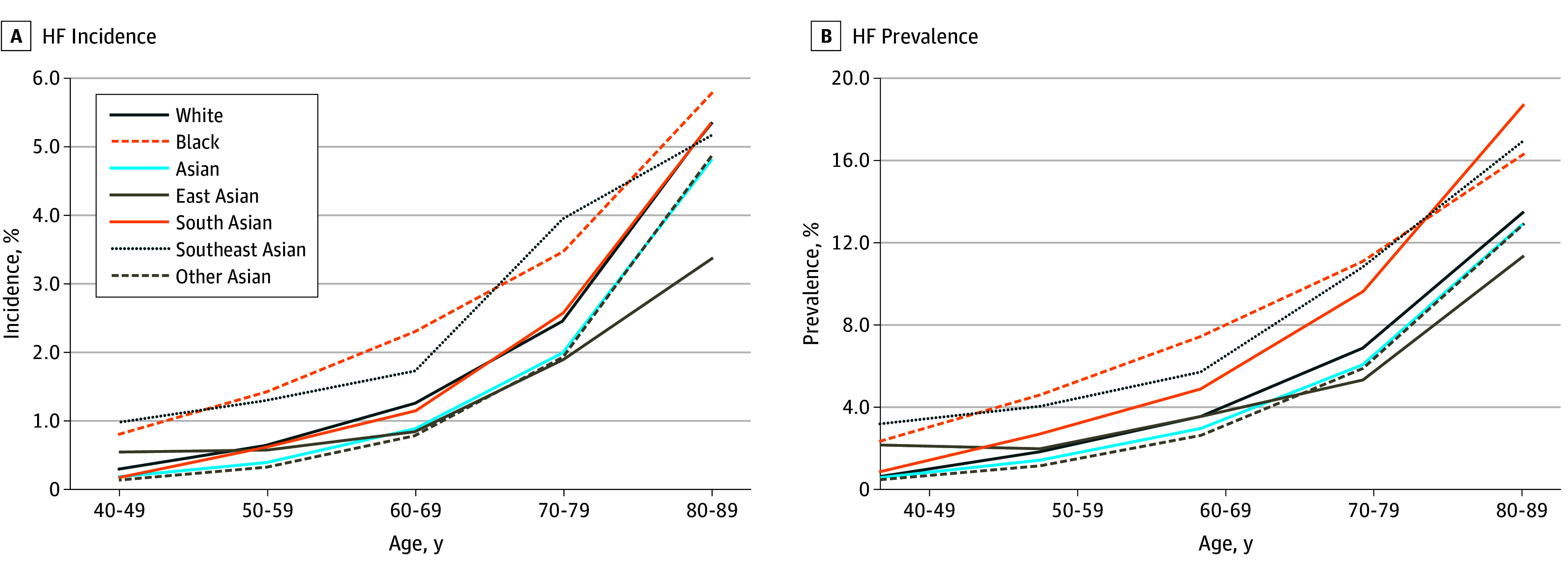
Incidence and Prevalence of Heart Failure (HF) by Age Categories for Race Other Asian indicates no other Asian ethnicity was specified.

The mean annual age-specific incidence and prevalence from 2015 to 2019 for Black, White, Asian, and Asian subgroup populations are shown in Figure 2. There were large differences among the different Asian subgroups, with Southeast Asian patients having the highest incidence of HF in most age groups, often exceeding those of White patients and close to those of Black patients. Differences in incidence between Southeast Asian and East Asian patients were notably pronounced compared with Black and White patients. Prevalence patterns generally mirrored incidence trends, with South and Southeast Asian patients in the highest age group (80-89 years) showing higher prevalence than Black patients.

Sex-stratified and age-standardized as well as age- and sex-standardized HF rates showed a lower incidence and prevalence among aggregated Asian patients (incidence, 1.29%; 95% CI, 1.26%-1.32%; prevalence, 3.85%; 95% CI, 3.81%-3.89%) compared with Black patients (incidence, 2.39%; 95% CI, 2.36%-2.42%; prevalence, 7.29%; 95% CI, 7.26%-7.33%) and White patients (incidence, 1.58%; 95% CI: 1.57%-1.59%; prevalence, 4.23%; 95% CI, 4.23%-4.24%) (Table 2). However, ethnicity-specific analysis revealed disparities, with Southeast Asian patients having the highest HF incidence (2.26%; 95% CI, 2.07%-2.45%) and prevalence (6.84%; 95% CI, 6.59%-7.08%), followed by South Asian (incidence, 1.56%; 95% CI, 1.31%-1.82%; prevalence, 5.86%; 95% CI, 5.49%-6.22%), East Asian (incidence, 1.22%; 95% CI, 1.06%-1.38%; prevalence, 4.19%; 95% CI, 3.98%-4.41%), and other Asian (incidence, 1.24%; 95% CI, 1.20%-1.27%; prevalence, 3.64%; 95% CI, 3.60%-3.68%) patients.

**Table 2.** Sex-Stratified and Age-Standardized and Age- and Sex-Standardized Incidence and Prevalence of Heart Failure^a^

Race group	Age-standardized rate, % (95% CI)		
	Female	Male	Age and sex stratified
**Incidence**			
White	1.38 (1.38-1.39)	1.98 (1.97-1.99)	1.58 (1.57-1.59)
Black	2.12 (2.09-2.15)	2.94 (2.89-3.00)	2.39 (2.36-2.42)
Asian	1.06 (1.03-1.10)	1.76 (1.69-1.82)	1.29 (1.26-1.32)
East Asian	0.99 (0.81-1.71)	1.70 (1.38-2.03)	1.22 (1.06-1.38)
South Asian	1.48 (1.15-1.81)	1.72 (1.32-2.13)	1.56 (1.31-1.82)
Southeast Asian	1.90 (1.68-2.12)	3.00 (2.64-3.37)	2.26 (2.07-2.45)
Other Asian^b^	1.02 (0.98-1.06)	1.68 (1.61-1.74)	1.24 (1.20-1.27)
**Prevalence**			
White	3.69 (3.69-3.70)	5.17 (5.16-5.18)	4.23 (4.23-4.24)
Black	6.62 (6.57-6.66)	8.48 (8.42-8.54)	7.29 (7.26-7.33)
Asian	3.14 (3.10-3.19)	5.10 (5.00-5.15)	3.85 (3.81-3.89)
East Asian	3.37 (3.13-3.61)	5.62 (5.22-6.02)	4.19 (3.98-4.41)
South Asian	5.35 (4.88-5.82)	6.74 (6.17-7.32)	5.86 (5.49-6.22)
Southeast Asian	5.67 (5.36-5.99)	8.86 (8.47-9.25)	6.84 (6.59-7.08)
Other Asian^b^	3.01 (2.96-3.06)	4.47 (4.66-4.82)	3.64 (3.60-3.68)

^a^ The 2015 population was used as the standard population.

^b^ Other Asian indicates no other Asian ethnicity was specified.

Table 3 highlights HF incidence and prevalence disparities. Compared with White patients, Black and Southeast Asian patients had a higher risk of developing HF, whereas East Asian and other Asian patients had a lower risk. Black, South Asian, and Southeast Asian patients had significantly higher HF prevalence than White patients, whereas other Asian patients had significantly lower rates.

**Table 3.** Risk Ratios of Age- and Sex-Standardized Incidence and Prevalence of Heart Failure by Race Groups

Race group	Risk ratio (95% CI)	
	Incidence	Prevalence
White	1.00 [Reference]	1.00 [Reference]
Black	1.51 (1.49-1.53)	1.72 (1.71-1.73)
East Asian	0.77 (0.67-0.89)	0.99 (0.94-1.05)
South Asian	0.99 (0.81-1.21)	1.38 (1.29-1.49)
Southeast Asian	1.43 (1.30-1.57)	1.61 (1.55-1.68)
Other Asian^a^	0.78 (0.76-0.80)	0.86 (0.85-0.87)

^a^ Other Asian indicates no other Asian ethnicity was specified.

## Discussion

Previous work has documented HF rate differences in Asian American groups,^21^ but this study is the first, to our knowledge, to use a large national EHR dataset to examine HF incidence and prevalence in 3 major Asian American subgroups. Although differences in HF rates between Black and White individuals are well known, disparities among Asian American subgroups have been less studied. Our analyses also highlight comparable HF incidence and prevalence between Southeast Asian American and Black American populations, a finding not previously reported.

Among the Asian American subgroups, our analysis indicates Southeast Asian American patients had the highest incidence and prevalence of HF across most age groups, followed by South Asian American patients, who notably exhibited the highest prevalence in older age groups. This finding is consistent with the limited evidence on HF mortality and hospitalization. When comparing Asian American subgroups, the highest age-standardized HF mortality rates have been found among the Indian American population.^21^ Filipino American patients compared with White American patients also have higher rates of preventable hospitalizations for HF,^29^ and Chinese American patients have higher 1-year mortality rates after HF hospitalization.^30^ Our study underscores significant disparities in CVD prevalence among East, South, and Southeast Asian American populations when data are disaggregated, highlighting critical health disparities within Asian subgroups.

Our findings echo prior research that highlights concerns about the unrecognized burden of HF across diverse Asian subgroups. Systemic barriers, including insurance status, financial challenges, and immigration history, may contribute to these disparities.^31,32,33^ Although the proportion of immigrants is not readily available information from EHR data, approximately 66% of Asian American people are born outside the US,^15,16,34^ which could affect HF rates due to factors such as unhealthy weight, poor diets, and reduced physical activity levels.^33^ Acculturation in the US context has also been linked to increased CVD risk among Asian American populations.^17^ These disparities are often masked in aggregated data: the aggregated Asian American group had a lower incidence and prevalence of HF compared with the White and Black American groups, probably driven by the lower HF rates in the East Asian American population.

The underlying CVD risk factors contribute to increased HF risk and high mortality among Asian American people.^35,36,37,38,39,40^ The unique sociocultural and environmental contexts experienced by Asian American people in the US should be acknowledged alongside genetic predispositions highlighted by the Asian Sudden Cardiac Death in Heart Failure study.^14^ Higher smoking rates among Asian American men and increasing rates among women as they acculturate have also been noted.^40^ These factors collectively contribute to a significant burden of CVD, including HF, among Asian American people. Effective long-term prevention and management strategies are crucial, considering the influence of environmental factors, such as dietary changes and neighborhood safety, on physical activity.

### Implications

The clinical implication of our study is that risk assessment and diagnostic decisions relating to HF should not regard the Asian American population as a monolithic group but instead consider individual Asian ethnicities and their respective cardiovascular risk factors. Previous aggregated data on HF outcomes have shown conflicting results.^19,41,42^ Health and health outcomes are often affected by both social determinants and genetics. Genetic data are often unavailable, and race is a social construct and a poor proxy for genetics. Limited disaggregated data on Asian American populations hinder nuanced health disparity studies despite our use of such data in this study. This limitation underscores the need for detailed documentation of ethnicity for Asian American, Native Hawaiian, and other Pacific Islander populations. Similar disaggregation approaches have been beneficial for other racial and ethnic groups, such as Hispanic/Latino and Black populations, in identifying health disparities and promoting equity.^43,44^ Previous studies often overlooked Asian American, Native Hawaiian, and Pacific Islander populations or categorized them as “other,” impeding meaningful health disparity insights due to their small sample sizes in datasets, such as the Framingham Heart Study^45^ and Cardiovascular Health Study,^46^ used for CVD analyses, including HF.

Asian American people are the fastest-growing US racial and ethnic subgroup with significant health disparities, which should draw greater attention to the need for further exploration together with the understanding of the unique social and structural determinants of health experienced by Asian American community members. New data collected on Asian American, Native Hawaiian, and Pacific Islander populations should be disaggregated by ethnicity because these populations are extremely diverse and aggregation can completely obscure the disparities. Our findings support the generalizability of Oracle EHR RWD as a source to evaluate national-level patterns of HF incidence and prevalence.

### Future Work

Future work is needed to collect data on HF and other CVDs from Asian American ethnic subgroups to address knowledge gaps and understand health disparities and outcomes influenced by social and structural determinants of health. Additional studies are needed to assess the quality of HF and CVD care and outcomes specific to Asian American, Native Hawaiian, and Pacific Islander populations, including risk factors, treatment patterns, and outcomes across different subgroups. Currently, reasons for high CVD risk in Asian American, Native Hawaiian, and Pacific Islander communities are not well understood, and further research is needed to examine barriers to care. Enhancing cohort studies and surveys with detailed ethnicity information and actively recruiting Asian American, Native Hawaiian, and Pacific Islander participants are essential steps, recognizing limitations in EHR data. Additionally, leveraging larger datasets, such as All Of Us, that include genetic data offers opportunities to explore HF and other CVDs in Asian American, Native Hawaiian, and Pacific Islander populations based on genetically determined ethnicity.

### Limitations

This study has some limitations. Despite the RWD being a large database, most of its records lack detailed ethnicity data for Asian American populations, which limits research opportunities. Asian patients with missing ethnicity information had lower estimates of HF incidence and prevalence, resembling the estimates of East Asian patients more closely than other ethnic groups. These lower estimates can be attributed to their lower rate of comorbid conditions such as CHD, acute MI, hypertension, diabetes, and stroke, similar to the East Asian patients and in contrast to the South Asian and Southeast Asian groups. In addition, Asian patients with missing ethnicity information showed a demographic distribution similar to East Asian patients.

Only 20 of 121 source facilities in the RWD database offer nuanced ethnicity data on Asian American populations. Given our focus on the incidence and prevalence of HF, we did not have a large enough sample size to disaggregate further than East, South, and Southeast Asian subgroups, yet our findings suggest further research and disaggregation are needed despite these limitations.

The RWD population changes over time, with relatively short follow-up periods (mean, 3-4 years), limiting the determination of patient history. Therefore, our analysis focused on active patients with recent visits to accurately assess incidence. The HF *ICD-9-CM* and *ICD-10-CM* codes are not always reliable. The inpatient diagnosis is more precise but not sensitive, whereas the outpatient diagnosis is sensitive but not precise. Nevertheless, we are comparing the HF rates across different racial groups using the same codes and the same standard population to estimate age- and sex-standardized rates.

For the incidence analysis, we included patients with encounters in the year preceding the study year. This approach may incur selection bias by excluding less frequent health care users. However, this approach aimed to avoid misclassifying newly diagnosed HF cases that might have been diagnosed elsewhere.

## Conclusions

Our analyses revealed significant disparities in HF incidence and prevalence among Asian American subpopulations. The HF rates among Southeast Asian American patients are similar to those of Black American patients, whereas the HF rates among East Asian American patients are less than those of White patients. This work highlights the urgent systemic need for collecting and analyzing disaggregated data on Asian American health disparities.

## Notes

Supplement.
Data Sharing Statement
