# Supplementary material for: Heart Failure Among Asian American Subpopulations
Source: JAMA Netw Open. 2024 Sep 26;7(9):e2435672. doi: 10.1001/jamanetworkopen.2024.35672 (PMC11428018; doi:10.1001/jamanetworkopen.2024.35672)
Supplement: Supplement. — Data Sharing Statement [file jamanetwopen-e2435672-s001.pdf]

Data Sharing Statement

Cheng. Heart Failure Among Asian American Subpopulations. *JAMA Netw Open*. Published October 01, 2024. doi:10.1001/jamanetworkopen.2024.35672

Data
Data available: No
